# Barriers to Accurate Diagnosis of Infantile Atopic Dermatitis: Insights From a Survey of Pediatricians

**DOI:** 10.1111/1346-8138.70052

**Published:** 2025-11-14

**Authors:** Kiwako Yamamoto‐Hanada, Yasusuke Kawada, Kana Okamoto, Miyuki Matsukawa, Takahiro Tsuchiya, Daisaku Michikami, Yukihiro Ohya

**Affiliations:** ^1^ Allergy Center National Center for Child Health and Development Tokyo Japan; ^2^ Kawada Children and Allergy Clinic Shizuoka Japan; ^3^ Department of Medical Affairs Otsuka Pharmaceutical Co. Ltd. Tokyo Japan; ^4^ Department of Occupational and Environmental Health Nagoya City University, Graduate School of Medical Sciences Aichi Japan; ^5^ Division of General Allergy, Bantane Hospital Fujita Health University Aichi Japan

**Keywords:** atopic dermatitis, diagnosis, early intervention, eczema, infant, Japan, pediatrician, questionnaire, survey

## Abstract

Accurate diagnosis is essential for timely intervention in atopic dermatitis (AD), yet delays in diagnosis remain common. To better understand current clinical practices regarding infantile AD, a questionnaire survey was conducted among Japanese pediatricians working at medical institutions with 19 or fewer beds. Respondents who provided informed consent completed an online questionnaire that included items on screening practices, physician background, understanding of diagnostic and treatment practices, and recognition of key clinical issues. In total, 238 valid responses were analyzed. Most respondents indicated that they were non‐allergists (85.7% of responses), aged 50 years or older (68.9% of responses) and reported high clinical experience in treating infantile eczema. Only 44.1% of respondents correctly recognized that AD is a condition within the collective term of infantile eczema. Almost all (92.0%) respondents correctly agreed that early intervention was effective for infantile AD and most recognized that AD treatment is prolonged, that AD induces other allergic diseases, and that AD is unlikely to resolve spontaneously in most cases. Understanding of the primary nature of AD was poor with 62.6% of respondents either incorrectly stating that AD is caused by other allergic diseases or that they did not know. The mean (SD) minimum age of AD diagnosis was 7.4 (4.81) months (median, 6.0 months) and 23.9% of physicians diagnosed AD after 1 year of age. Only 16.4% of respondents correctly identified a case of infantile AD and only 19.3% of respondents correctly selected the most appropriate treatment for a known case of infantile AD. Reluctance to inform parents/caregivers of an AD diagnosis was high and mostly due to anticipation of parental shock. Certain pediatricians in Japan have misunderstandings about infantile AD. Further awareness of infantile AD is necessary to ensure early diagnosis and intervention as well as management aligned with guideline recommendations.

## 1 Introduction

Infantile eczema is a broad, non‐specific term referring to a range of common inflammatory skin disorders in infancy, typically characterized by dry, pruritic, erythematous eruptions affecting the face and flexural areas [1]. Atopic dermatitis (AD), a chronic, immune‐mediated condition included in infantile eczema, may be misdiagnosed as infantile eczema [2]. This leads to a lack of appropriate treatment for AD because doctors tend to misunderstand that infantile AD and infantile eczema are separate diseases. In support of the issue of underdiagnosis of AD in infants, the Japan Environment and Children's Study showed that the number of infants diagnosed with AD is only 25%–50% of the prevalence calculated from symptoms reported by parents/caregivers according to the International Study of Asthma and Allergies in Childhood (ISAAC) Questionnaire [3, 4]. A cohort study of itchy infantile eczema in the general population also showed that about 80% of patients with itchy eczema had a diagnosis of infantile eczema and only 14% had a diagnosis of AD, which suggests that many infants with AD remain undiagnosed and not treated appropriately [5].

AD that develops in infancy may trigger percutaneous sensitization and constitute one of the causes of food allergy in childhood. Therefore, early intervention to treat infantile AD and inhibit percutaneous sensitization is expected to prevent ‘atopic march’, including food allergy [6, 7]. Diseases presenting with eczema, such as seborrheic and contact dermatitis, are likely to resolve relatively quickly in infancy [2]. In contrast, AD generally persists and may pose a risk of percutaneous sensitization via IgE and related immune mechanisms [8, 9]. The Japanese Guidelines for Food Allergy 2021 state that AD is particularly important as a risk factor for food allergy [10]. Delayed diagnosis of infantile AD prevents early resolution and leads to the onset of the atopic march. Early intervention of AD could prevent severe AD and subsequent egg allergy [11, 12].

Although delays in the diagnosis of AD have been increasingly recognized, little research has investigated the underlying barriers to accurate diagnosis or the reasons why physicians may hesitate to diagnose infantile AD [13, 14]. To address this gap, we conducted a questionnaire‐based survey among pediatricians working at medical institutions in Japan with 19 or fewer inpatient beds, aiming to elucidate current clinical practice challenges related to the diagnosis and management of infantile AD.

## 2 Methods

### 2.1 Study Design

This was a cross‐sectional observational study based on an internet questionnaire survey, conducted by Social Survey Research Information Co. Ltd. (Tokyo, Japan) among pediatricians, which collected responses broadly related to the current diagnosis and treatment of AD and infantile eczema, as well as recognition of topics related to these conditions. Eligible pediatricians were those working for a medical institution with ≤ 19 beds who had treated 10 or more children aged 0 years in the latest month and who routinely treat infantile eczema and/or infantile AD.

Doctors considered eligible, based on responses to screening items, were presented with a web‐based explanatory document on the study outline and handling of personal information. Doctors who agreed to the document were allowed to proceed to the survey by giving web‐based consent and were provided with a survey link by the medical information website m3.com (M3 Inc., Tokyo, Japan).

This study was approved by the research ethics committee of Kitamachi Clinic, Fujikei Medical Corporation on October 25, 2023 (Reception number OTH09785).

### 2.2 Investigation Items

The Questionnaire Form for Survey on Clinical Practice for Infantile Atopic Dermatitis summarizing this survey is included as Table S1. The full questionnaire is also included in the Supporting Information. In addition to the top‐down organization, this questionnaire can also be reorganized and broken down into sections and items related to:

- Screening (items F1‐3, S1‐2)
- Background information on doctors, including treatment policy for infantile AD and treatment experience of infantile eczema (Q1, Q3, Q10, F4‐6)
- Understanding of current status of diagnosis, including key issues presented in this report of recognition of infantile AD and infantile eczema (Q2), the age in month when a diagnosis of AD and infantile eczema is made (Q5, Q12) and reluctance to make a diagnosis of atopic dermatitis and its reason (Q18, Q19) (Q2, Q5, Q7, Q8, Q11, Q12, Q14, Q15, Q18, Q19)
- Understanding of current status of treatment, including drugs prescribed for AD and infantile eczema (Q9, Q16) as covered in this report (Q9, Q16, Q17, Q24‐27)
- Doctor's recognition of important topics, including understanding of the dual‐allergen exposure hypothesis (Q21) as covered in this report (Q4, Q21, Q28)

### 2.3 Sample Size

Being a descriptive study, the target sample size was not based on statistical power. Rather, considering the possibility of collecting the survey results, it was set at 240 doctors based on a previous survey in a related condition conducted by PatientsMap, a joint medical care patient database of Social Survey Research Information Co. Ltd. (SSRI) and M3 Inc. (a medical information website), which was both used in the current study.

### 2.4 Statistical Analysis

For categorical variables, summary statistics, including the number of responses and composition ratio (%), were calculated. For continuous variables, response counts, mean, standard deviation, minimum, maximum, median, 25 and 75 percentiles, interquartile range, and 95% confidence interval (CI) values were calculated.

Data compilation was performed using BellCurve Hideyoshi Dplus (version 1.13) and statistical analysis was performed using BellCurve for Excel (Social Survey Research Information Co. Ltd., Tokyo, Japan).

## 3 Results

### 3.1 Survey Respondent Flow and Characteristics

**FIGURE 1:**
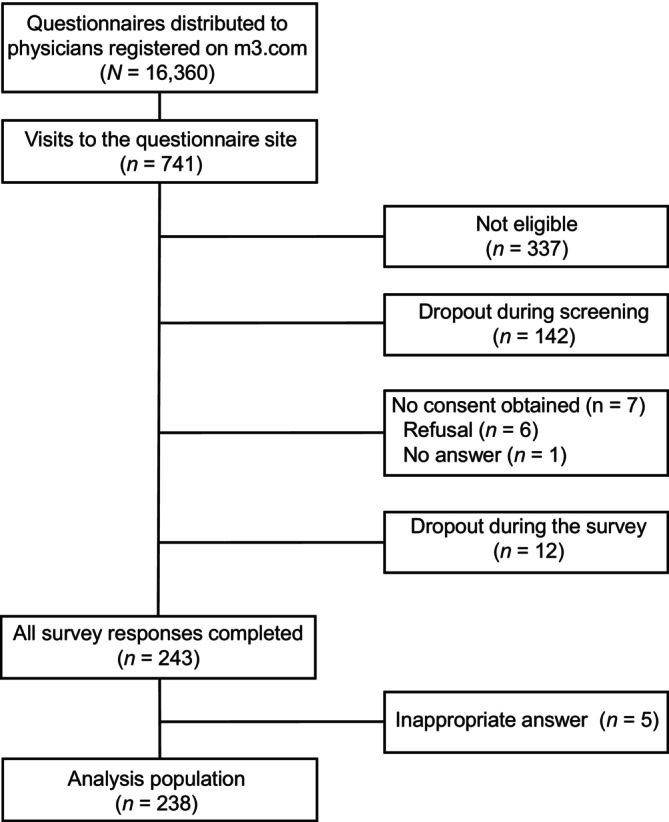
Survey respondent flow.

During the survey period from October 27, 2023 to November 26, 2023, a total of 16 360 questionnaires were distributed to physicians registered on the m3.com site and 741 visits to the site were registered. According to the survey target flow, a total of 243 survey responses were completed and 238 responses comprised the analysis population after exclusions for ineligibility, dropouts, lack of obtained consent and inappropriate responses (Figure 1).

The characteristics of the survey respondents, as shown in Table 1, reveal that 85.7% were non‐allergists and 68.9% were aged 50 years or older. Clinical experience with treating infantile eczema was reportedly high, with 53.8% reporting 10–29 years of clinical experience and 38.7% reporting 30 years or more.

**TABLE 1:** Characteristics of survey respondents.

		*N*	%
Number of beds	0	224	94.1
	1–19	14	5.9
Japanese Society of Allergology specialist or attending physician	Japanese Society of Allergology specialist	31	13.0
	Japanese Society of Allergology attending physician	6	2.5
	None	204	85.7
Age (years old)	20s	—	—
	30s	18	7.6
	40s	56	23.5
	50s	69	29.0
	60s	79	33.2
	≥ 70	16	6.7
Number of 0‐year‐old children treated (per month)	0	—	—
	1–99	119	50.0
	100–199	64	26.9
	200–299	28	11.8
	300–399	12	5.0
	≥ 400	15	6.3
Clinical experience of infantile eczema (years)	< 10	14	5.9
	10–19	60	25.2
	20–29	68	28.6
	≥ 30	92	38.7
	Unknown	4	1.7

### 3.2 Awareness of the Relationship Between Infantile AD and Eczema

**FIGURE 2:**
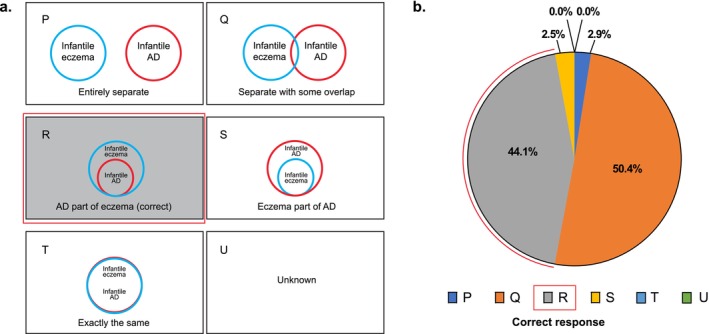
Awareness of relationship between infantile AD and infantile eczema including (a) relationship scenarios physicians could select from (b) proportion of responses for each scenario.

When presented with Venn diagrams showing possible relationships between infantile AD and infantile eczema (Figure 2a), 44.1% of respondents correctly recognized the relationship that AD is a condition within the collective term of infantile eczema (Figure 2b). In contrast, half of respondents incorrectly viewed infantile AD and infantile eczema as mostly separate conditions with the potential for overlap in some patients.

### 3.3 Awareness of Infantile AD and the Dual Antigen Exposure Hypothesis

Overall responses and the proportion of correct responses to a range of questions to assess awareness of AD and the dual antigen exposure hypothesis are shown in Table 2. Almost all (92.0%) respondents correctly agreed that early intervention was effective for infantile AD. Similarly, most respondents recognized that AD treatment is prolonged, that AD induces other allergic diseases, and that AD is unlikely to resolve spontaneously in most cases. In contrast, while 90.8% of physicians correctly understood the importance of percutaneous sensitization, only 43.7% of physicians were able to correctly identify that the statement “Sensitization is established by the oral intake of allergen” is incorrect. Further, understanding of the primary nature of AD was poor with most (62.6%) respondents either incorrectly stating that AD is caused by other allergic diseases or that they did not know.

**TABLE 2:** Responses to questions regarding understanding of infantile AD diagnosis and management.

Questionnaire	Agree (%)	Don't agree (%)	Don't know (%)	Proportion with correct answer (%)
Early therapeutic intervention is effective	92.0	4.2	3.8	92.0
Sensitization to allergens is established percutaneously	90.8	4.2	5.0	90.8
Treatment is given for a short period	4.6	89.9	5.5	89.9
It takes a long time to treat the disease	84.9	10.5	4.6	84.9
The disease induces other allergic diseases	80.3	13.4	6.3	80.3
The oral intake of allergens promotes immune tolerance	74.4	11.8	13.9	74.4
The disease resolves spontaneously in most cases	15.1	71.4	13.4	71.4
The intake of foods causing allergy, such as eggs, should be delayed	21.4	70.2	8.4	70.2
Eczema can be eliminated with appropriate treatment	62.6	25.2	12.2	62.6
Sensitization is established by the oral intake of allergen	40.3	43.7	16.0	43.7
The disease is caused by other allergic diseases	42.9	37.4	19.7	37.4

[Correction added on 11 December 2025 after first online publication: In Table 2, the second‐to‐last row has been moved to the second row from the top.]

### 3.4 Age at Diagnosis of Infantile AD

**FIGURE 3:**
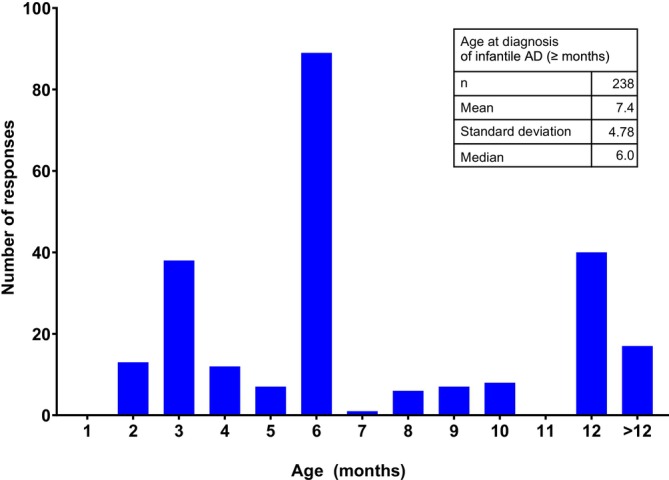
Minimum age at diagnosis of infantile AD.

When asked to specify the age in months of infants at or above which a diagnosis of AD is made, physicians answered 6 months, followed by 12 months. The mean (SD) minimum age was 7.4 (4.78) months (median, 6.0 months; Figure 3) and 23.9% of physicians responded that infantile AD was diagnosed after 1 year of age.

### 3.5 Diagnosis and Treatment of Specific Cases

**FIGURE 4:**
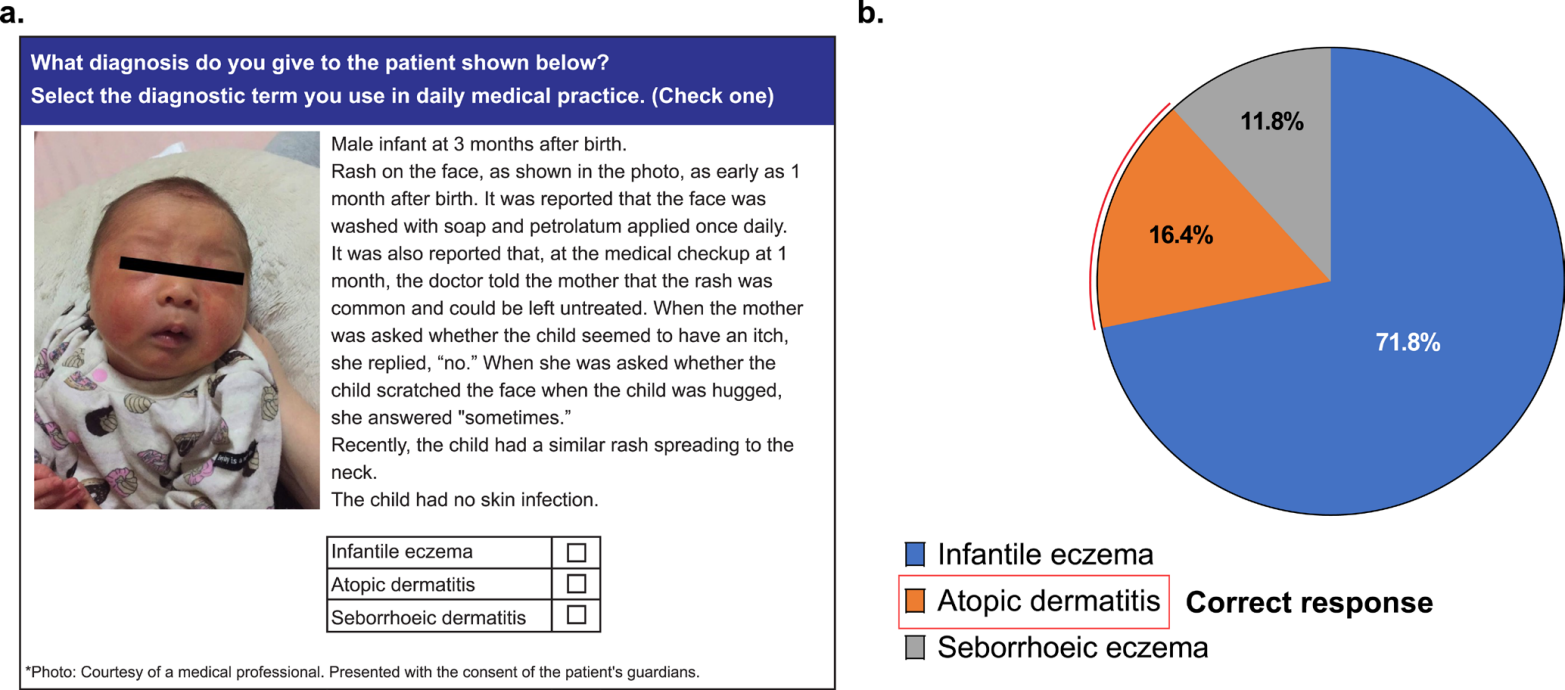
Diagnosis of a specific case including (a) case description and diagnosis options (b) proportion of physicians who selected each option.

Physicians were asked to provide a diagnosis based on the image and clinical history presented in Figure 4a (Q13 of web‐based survey in Supporting Information). Clinical features that matched the diagnostic criteria for infantile AD included itching (suggested by occasional scratching of the face when held in the case), the distribution of a characteristic skin rash (i.e., a similar rash also spreading to the neck in the case), and the chronic course of the condition (i.e., having begun and continued from 1 month of age in the case). However, despite these features, only 16.4% of respondents correctly selected infantile AD while 71.8% selected the broader term of infantile eczema (Figure 4b).

**FIGURE 5:**
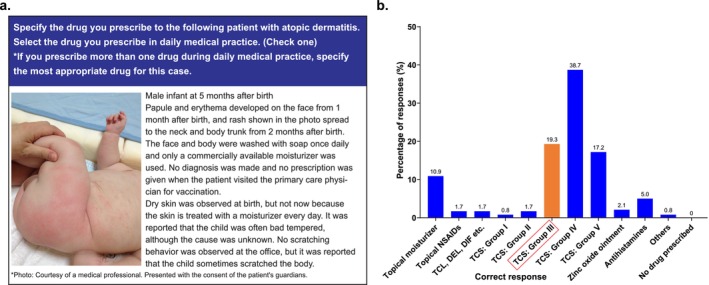
Prescribed drugs for a specific case including (a) case description (b) proportion of respondents selecting each possible single drug option.

Similarly, when asked to select one drug treatment most appropriate for an infant with AD described by the image and clinical history presented in Figure 5a (Q17 of web‐based survey in Supporting Information), the most common response was group IV steroids (38.7%) followed by group III steroids (19.3%); 10.9% of respondents chose a moisturizer without anti‐inflammatory properties (Figure 5b).

### 3.6 Resistance to Infantile AD Diagnosis

**FIGURE 6:**
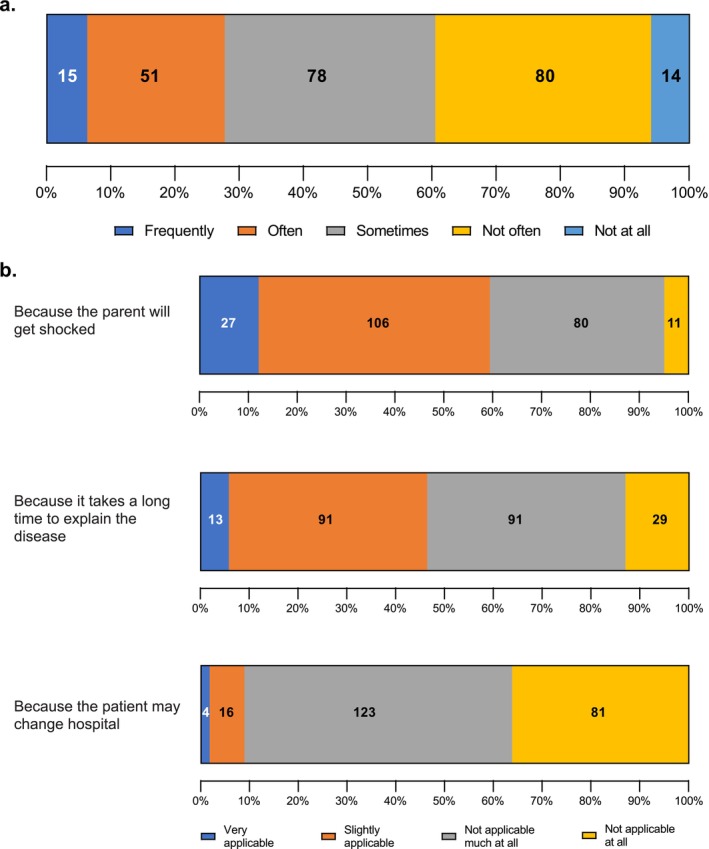
(a) Reluctance to diagnosing infantile AD and (b) proportion of agreement with reasons for the reluctance among respondents.

In total, 60.5% of respondents stated they frequently, often, or sometimes felt reluctant to tell the parent/caregiver of an infant with AD the diagnostic term in daily medical practice (Figure 6a) (Q18 of web‐based survey in Supporting Information). Regarding the reasons for this reluctance, the most common reason was anticipation of parental shock for which 59.4% of respondents regarded this as “very” or “slightly” applicable. Secondly, the reason of time taken to explain the disease was regarded by 46.4% of respondents as “very” or “slightly” applicable (Figure 6b).

## 4 Discussion

This is the first observational study using a questionnaire to physicians to investigate the awareness of relationships between infantile eczema and infantile AD. These findings suggest that there is a lack of understanding of the causes of infantile AD, its relationship to infantile eczema, diagnosis, and treatment. A proper understanding of infantile AD is important for appropriate and early diagnosis and intervention, and further awareness raising appears necessary based on reports of underdiagnosis of this condition.

Regarding the relationship between infantile AD and infantile eczema, results of the Venn diagram questionnaire suggest that less than half of Japanese physicians correctly understand that infantile AD is a subset of infantile eczema. In contrast, the dominant understanding of the relationship was that they are separate conditions with some overlap. In the UK, both infantile AD and infantile eczema are diagnosed as eczema so the separation of these conditions seems to be an issue unique to Japan.

Regarding the age of diagnosis, 92% of pediatricians surveyed were aware that early intervention is effective for infantile AD although the actual mean minimum age of diagnosis was 7.4 months. This is despite the fact that the onset of AD typically occurs in the early postnatal period [15, 16, 17, 18, 19], suggesting there may be differences between the time of onset and diagnosis in actual practice. We focused on the month of diagnosis because children start eating solid foods at 4–5 months of age, at which stage there is a risk of sensitization leading to the development of food allergies. A birth cohort study in Japan examined risk ratios for food allergies at 3 years of age for infants with and without eczema according to the age of eczema onset. The adjusted odds ratio was highest for infants with eczema onset at 1–2 months of age (adjusted odds ratio 6.61), followed by onset at 3–4 months (adjusted odds ratio 4.69) [20]. In a retrospective cohort study, it was also revealed that a longer period from the onset of AD to the initiation of treatment was associated with a higher prevalence of food allergy at 2 years of age [21]. Furthermore, enhanced early skin treatment for infants with early‐onset AD, who are at high risk of developing food allergy, has been shown to prevent the onset of food allergy in randomized controlled trials [12]. Based on the above evidence, it should be considered essential to have an early diagnosis of AD during infancy to prevent the onset of food allergy.

Based on case history results, the rate of appropriate diagnosis and drug selection was less than 20%. Although there were descriptions of pruritus and the distribution of characteristic eruptions in the case history, these features may have been overlooked by respondents. Alternatively, the age of 3 months might have influenced the diagnosis since the mean minimum age of AD diagnosis (7.4 months) was considerably older than that observed in previous observational studies in Japan [17]. Application of the U.K. Working Party's Diagnostic Criteria for AD [22], which requires one major criterion and 3 or more minor criteria to be satisfied, should ideally be used to achieve early diagnosis. The original version of these criteria has been validated in a study of Japanese elementary schoolchildren and been shown to have good sensitivity and specificity in this population [23].

We presented the case of a patient who needed treatment with a Group III topical steroid. Overall, physicians in this survey tended to select a topical corticosteroid less potent than that required by the case presented. Inadequate treatment of AD may lead to several adverse outcomes, including repeated relapses and the risk of percutaneous sensitization [24, 25]. A previous large‐scale questionnaire survey in the USA, UK, and Japan found that Japanese physicians were significantly less aggressive than Western counterparts in prescribing practices, including those related to topical corticosteroids [26]. This survey discussed the possibility of negative propaganda against topical corticosteroids in Japan as contributing to this discrepancy, which may also relate to a greater tendency among Japanese physicians to use alternative remedies such as Chinese herbal preparations [26].

The lack of accurate diagnosis of AD in infants may be due to physician reluctance to diagnose it, which was commonly noted among the respondents in this survey. A key reason physicians hesitate to diagnose AD is to avoid shock among caregivers. In the background of this reason, physicians and caregivers possibly misunderstand that AD is an incurable disease. With regard to this, it is necessary to confirm whether the result in this study, that “the most common reason for being reluctant to diagnose atopic dermatitis in infants was the anticipated shock that parents would experience”, is actually what parents feel. If parents generally wish to be told the correct diagnosis, then education of physicians is necessary; whereas, if parents generally feel even more shocked, then research into the reasons for this, the spread of new treatments, and education about the disease are necessary. Regardless of the result, one way to alleviate such concerns among physicians would be to educate physicians and patients at academic conferences, seminars, and similar forums that communicating the correct diagnosis and implementing appropriate treatment in accordance with a diagnosis of atopic dermatitis early on leads to early remission. Also, it is necessary to change this misperception in line with the development of management guidelines and increase in treatment options. Further, it is possible that the reluctance to diagnose infantile AD relates to a lack of confidence in treatment aspects of management. Since this was a survey of physicians, we did not investigate the attitudes of parents and caregivers to the diagnosis and treatment of AD. There may be a gap between the perceptions of medical professionals and parents of patients, and it is necessary to investigate this aspect of actual practice.

This study was conducted with pediatricians in facilities with 19 beds or less registered with the physician panel owned by m3 Corporation. The results may have differed if pediatricians with specialized experience and knowledge from large hospitals or specialized centers were included. Further, because this study was conducted on physicians who use the internet, there is a possibility of selection bias to include physicians who respond positively to the survey. We acknowledge that both of these issues limit the generalizability of the study findings. The survey items in this study were based mainly on responses to a questionnaire survey. Since these were self‐answered, there is a possibility of recall bias and self‐report bias.

In conclusion, some pediatricians in Japan have a wrong understanding of infantile AD in terms of causality, the relationship to infantile eczema, diagnosis, and treatment strategies. By achieving the right understanding of infantile AD, physicians can provide appropriate treatment. As awareness of infantile AD increases, it is possible that early diagnosis and intervention as well as appropriate management in line with guideline recommendations will ensue.

## Ethics Statement

This study was approved by the research ethics committee of Kitamachi Clinic, Fujikei Medical Corporation on October 25, 2023 (Reception number OTH09785). As this was a questionnaire‐based survey of physicians, no consent to participate from patients was required.

## Consent

Completion of the questionnaire was regarded as implied informed consent. All responses were anonymous, and participants agreed to the publication of aggregated results for academic purposes.

## Conflicts of Interest

Kiwako Yamamoto‐Hanada has received funding research from Otsuka Pharmaceutical Co. Ltd.; consultancy or commissioned fees from Otsuka Pharmaceutical Co. Ltd., Sanofi K.K., and AbbVie G.K.; a fellowship, research grant or education grant from Kao Corporation, Natural Science Co. Ltd., Alcare Co. Ltd., and Fam's baby Inc.; speaking fees from Otsuka Pharmaceutical Co. Ltd., Torii Pharmaceutical Co. Ltd., Sanofi K.K., Regeneron, AbbVie, and Pfizer. Yasusuke Kawada has received consulting fees from Otsuka Pharmaceutical Co. Ltd.; speaking fees from Otsuka Pharmaceutical Co. Ltd., Maruho Co. Ltd., Torii Pharmaceutical Co. Ltd., Sanofi K.K., AbbVie G.K., and Shionogi and Co. Ltd.; conference participation fees, travel expenses, and accommodation expenses from Otsuka Pharmaceutical Co. Ltd. Kana Okamoto, Miyuki Matsukawa, Takahiro Tsuchiya, and Daisaku Michikami are all employees of Otsuka Pharmaceutical Co. Ltd. Yukihiro Ohya has received consultancy or commissioned fees from AbbVie, LEO Pharma, Maruho Co. Ltd., Otsuka Pharmaceutical Co. Ltd., and Sanofi K.K.; speaking fees from AbbVie, Kyorin Pharmaceutical Co. Ltd., LEO Pharma, Lilly, Maruho Co. Ltd., Otsuka Pharmaceutical Co. Ltd., Pfizer, Sanofi K.K., Regeneron, Taiho Pharmaceutical Co. Ltd., Torii Pharmaceutical Co. Ltd.

## Supporting information


**Table S1:** Overview of survey.

## Data Availability

The data that support the findings of this study are available from the corresponding author upon reasonable request.
