# Supplementary material for: Barriers to Accurate Diagnosis of Infantile Atopic Dermatitis: Insights From a Survey of Pediatricians
Source: J Dermatol. 2025 Nov 14;53(3):421–9. doi: 10.1111/1346-8138.70052 (PMC12967741; doi:10.1111/1346-8138.70052)
Supplement: Supplementary file 1 — Table S1: Overview of survey. [file JDE-53-421-s001.docx]

**Table S1.** Overview of survey

|  | **Screening items** |
| --- | --- |
| F1 | Institution management style |
| F2 | Number of beds |
| F3 | Responsible department |
| S1 | Number of pediatric patients examined |
| S2 | Availability of treatment for infantile skin disease |
|  | **Survey items** |
|  | Treatment status for AD and infantile eczema |
| Q1 | Number of doctors at the institution |
| Q2 | Recognition of AD and infantile eczema (Venn diagram) |
| Q3 | Number of patients with infantile eczema or infantile atopic dermatitis examined |
|  | Infantile eczema |
| Q4 | Diagnosis of infantile eczema |
| Q5 | Age in month when the diagnosis of infantile eczema is made |
| Q6 | Explanation to parents/caregivers about infantile eczema |
| Q7 | Degree of difficulty in making a diagnosis of each skin disease in daily clinical practice |
| Q8 | Diagnostic term of each skin disease in daily clinical practice |
| Q9 | Drugs prescribed to patients with infantile eczema |
|  | Infantile atopic dermatitis |
| Q10 | Treatment policy for infantile atopic dermatitis (treatment at the institution or reference to another institution) |
| Q11 | Point to emphasize in making a diagnosis of infantile atopic dermatitis |
| Q12 | Age in month when the diagnosis of infantile atopic dermatitis is made |
| Q13 | Diagnostic terms of specific cases |
| Q14 | Explanation to parents/caregivers |
| Q15 | Reactions of parents/caregivers |
| Q16 | Drugs prescribed to patients with infantile atopic dermatitis |
| Q17 | Drugs prescribed to specific cases |
| Q18 | Reluctance in making a diagnosis of AD |
| Q19 | Reasons for the reluctance in making a diagnosis of AD |
| Q20 | Frequency of explaining infantile eczema to parents/caregiver of patients with AD |
| Q21 | Recognition of AD/double antigen exposure hypothesis (degree of agreement) |
| Q22 | Expertise on atopic dermatitis |
| Q23 | Confidence in treating atopic dermatitis |
|  | External preparations |
| Q24 | Person who explains the application method and contents of explanation |
| Q25 | Contents of explanation on application method and explanatory materials |
| Q26 | Time required to explain application method |
| Q27 | Things to do to decide on treatment policy for return patients |
| Q28 | Thinking on the use of topical corticosteroids in infants |
|  | Attribute information |
| F4 | Specialist/advisory doctor authorized by the Japanese Society of Allergology |
| F5 | Age |
| F6 | Experience in the treatment of infantile eczema |

**Questionnaire on Pediatric Treatment**

| [Subject conditions]  ○ Pediatrician who works for a medical institution with ≤ 19 beds  ○ There are several other conditions  [Scope of disclosure, etc.] In conformity to the Act on the Protection of Personal Information and its guidelines, only the aggregation results not containing the identifying information on respondents are disclosed to the following degree of disclosure (■).  □ 1. Disclosed to our company and sponsor of this survey.  □ 2. May be disclosed to healthcare professionals under the confidentiality agreement intended for the advisory services to the sponsor of this survey, and to related ministries/agencies.  □ 3. May be disclosed to m3.com members (or within the m3.com site).  □ 4. May be disclosed only to healthcare professionals.  ■ 5. May be disclosed to the public.  In addition, we will process questionnaire respondents as follows where necessary to share individual responses, aggregation results, and analytical results with third parties to the extent that respondents cannot be identified.  🞄 Based on the classification specified by us or the third party, respondents of this questionnaire are confronted with the classification.  🞄 The respondents of this questionnaire are confronted with the responses of prior related questionnaires.  [Response method]  The response entered in any page of the questionnaire is automatically saved even when the browser is closed before finishing the questionnaire and the response to the residual questions can be resumed at the next access, provided that the deadline of the questionnaire is not overdue. |
| --- |

Now select the following and click "Next" to continue.

○ Pediatrician who works for a medical institution with ≤ 19 beds

Next

**First, let us know your primary place of work to statistically analyze your responses.**

| **Face sheet** | |
| --- | --- |
| F1. Institution management style  (Check one) | 1. University hospital  2. Other national/public hospital  3. General hospital (hospital other than the above)  4. Clinic/practitioner  5. Others |
| F2. Number of beds  (Check one) | 1. 0 beds  2. 1 to 19 beds  3. 20 to 99 beds  4. 100 to 199 beds  5. 200 to 499 beds  6. 500 beds or more |
| F3. Responsible department  (Check one) | 1. Pediatrics  2. Internal medicine (general internal medicine)  3. Otorhinolaryngology  4. Department of Allergy  5. Others |

Go to NEXT if F1 is "Clinic/practitioner" AND F2 is "0 beds" or "1 to 19 beds" AND F3 is "Pediatric Department.”

Screen out respondents who chose the other choices.

**First, let us know the current status of the clinical practice for pediatric patients.**

**S1**

Specify the actual number of pediatric patients you examined/treated in the latest 1 and 3 months on the medical record basis

(enter numerical value).

**Answer based on the status at your main workplace you answered above.**

|  | Latest **1 month**  (actual number based on medical records) | Latest **3 months**  (actual number based on medical records) |
| --- | --- | --- |
| 0 years | (n) | (n) |
| 1 to 5 years | (n) | (n) |

--- Page break

For the above description, screen out the responders who answered that the number of patients aged 0 years in the latest month was < 10.

**S2**

Of the following diseases, specify all the disease(s) you treat in daily medical practice.

(check all that apply)

| Infantile eczema | □ |
| --- | --- |
| Contagious impetigo | □ |
| Infantile atopic dermatitis | □ |
| Hand-foot-and-mouth disease | □ |
| Varicella | □ |
| Infectious erythema | □ |
| Other than the above | □ |

For the above description, screen out the responders who checked neither of infantile eczema or infantile atopic dermatitis (include those who checked either or both of them in this survey).

[Sponsor of survey] Otsuka Pharmaceutical Co., Ltd.

[Survey company] Social Survey Research Information Co., Ltd.

**Survey on Clinical Practice for Infantile Atopic Dermatitis**

This survey consists of the statement of request and questions. It takes about 20 minutes to complete the questions.

[Precautions]

If you agree to cooperate with the survey after understanding the following precautions, please check the section of "I agree" below.

🞄 Purpose of this survey: This survey is intended to ask doctors involved in the clinical practice for pediatric patients with dermal diseases about the thinking on the treatments and examinations for infantile atopic dermatitis.

🞄 The survey results including your responses will be used only for a research purpose, statistically processed in a way that prevents personal identification, and published in presentations or papers at academic conferences. No information identifying individuals will be disclosed.

🞄 This survey is conducted from October 27, 2023 to November 26, and the data obtained will be used for analysis.

🞄 This survey is conducted by M3, Inc. which is the subcontractor of the survey company under the request from the survey sponsor. The survey sponsor bears all the costs required for the survey. The Ethics Committee has reviewed and approved in advance that there is no conflict of interest between the survey sponsor and survey contractor that may compromise the reliability of the survey, and that the survey is conducted under an appropriate implementation system.

🞄 The intellectual property rights arising from this survey belong to the survey sponsor.

🞄 The survey results can be provided to regulatory authorities and ethical committee as necessary in a way that prevents personal identification.

🞄 Social Survey Research Information Co., Ltd. is responsible for the management of the obtained data.

🞄 You are not forced to participate in this survey. Whether or not to answer the questionnaire is up to you.

🞄 You can stop answering questions any time during the survey.

🞄 You will not have any disadvantage even if you do not answer questions or stop answering questions.

🞄 Your responses are strictly protected through the processing, data storage, and disposal stages. Your personal information will not leak outside.

Contact the following if you wish to withdraw your study consent or if you have any other questions.

Social Survey Research Information Co., Ltd.

e-mail: info3@ssri.co.jp

Japan Marketing Research Association Member No. 20111

Survey manager: Hishita, HBI/MA Bureau

Data management: Miura, HBI/MA Bureau

If you check "I agree", proceed to the questions starting on the next screen and answer questions.

I agree ○ I do not agree ○

If you agree, proceed to the next screen.

**These are questions about the clinical practice for skin diseases in infants.**

Let us know the number of doctors involved in medical treatment at your institution and that of doctors involved in the treatment of skin diseases in infants.

(enter numerical value).

*** Include yourself in your answers.**

| Number of doctors involved in medical treatment at your institution (including part-time doctors) | (n) |
| --- | --- |
|  | ↓ |
| Among them, the number of doctors involved in the treatment of skin diseases in infants | (n) |

"0" cannot be entered in the upper row.

Upper row ≥ Lower row

Select the image of infantile atopic dermatitis and eczema that is closest to your recognition from the figure below.

(Check one)


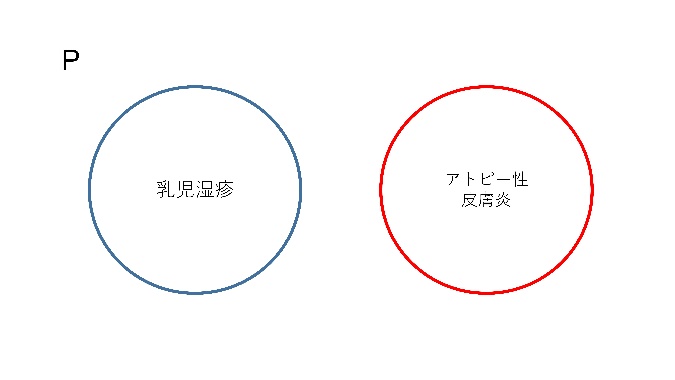

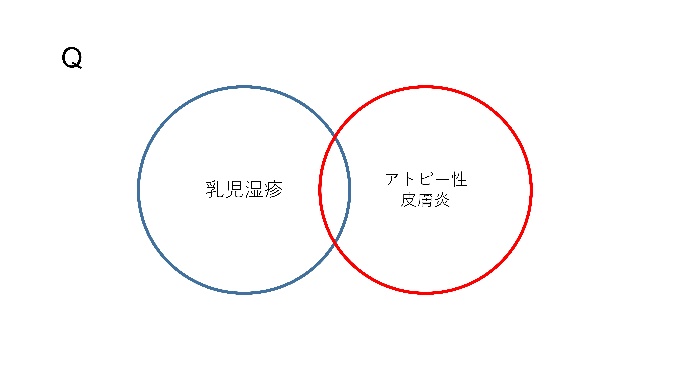

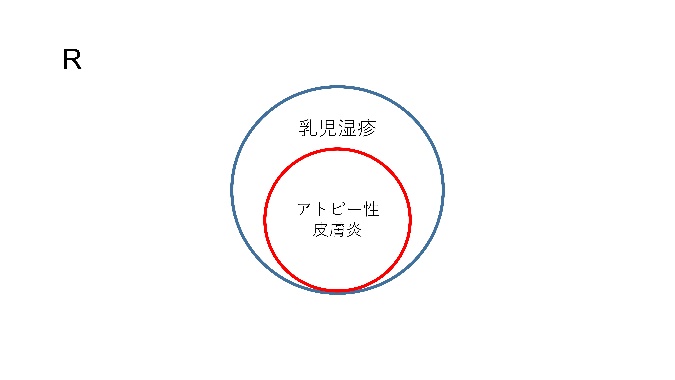

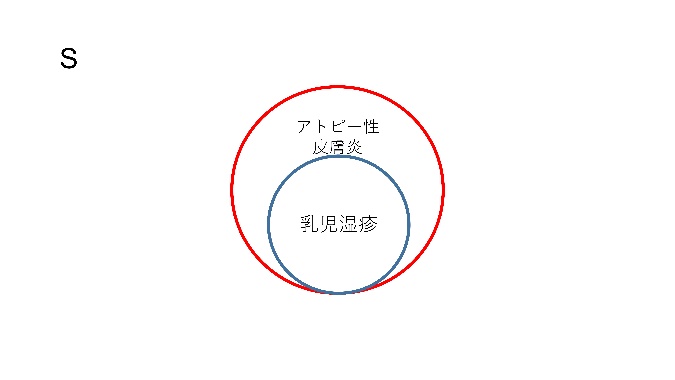

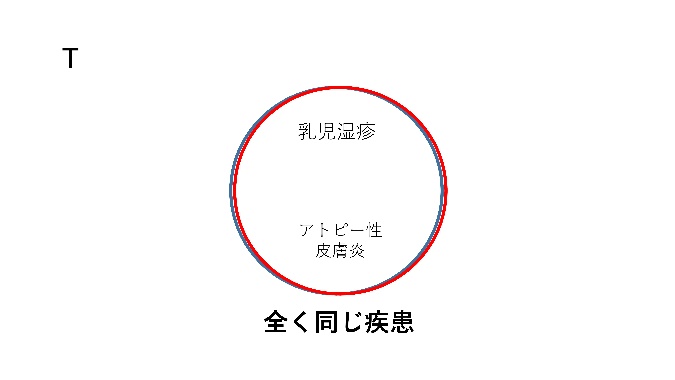

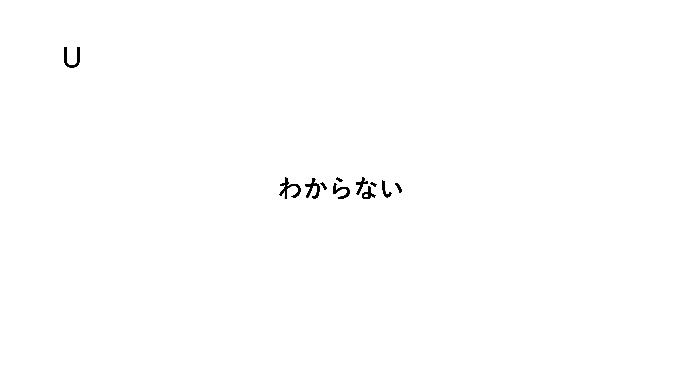


Infantile atopic dermatitis

Infantile eczema

Infantile eczema

Infantile atopic dermatitis

Infantile eczema

Infantile atopic dermatitis

Infantile eczema

Infantile atopic dermatitis

Infantile eczema

Exactly the same disease

Unknown

Infantile atopic dermatitis

--

Images are randomly arranged ("don't know" is fixed at the end)

Let us know the number of infantile patients with infantile eczema or atopic dermatitis treated in the latest 3 months.

(enter numerical value).

*** Enter zero when there is no such patient.**

The image selected in the previous question is reposted.

|  | In the latest 3 months |
| --- | --- |
| 0 years | S1 reposted (n) |
| *** Multiple answers** | ↓ |
| a) Infantile eczema | (n) |
| b) Infantile atopic dermatitis | (n) |
| c) Infantile eczema and infantile atopic dermatitis | (n) |

C) is displayed only to the respondents who selected the image of "Q" in the previous question.

Error if S1 reposted < each response value

**From here, let us ask about infantile eczema.**

Check all the answers close to your thinking for the diagnosis of "infantile eczema."

(check all that apply)

| Infantile eczema is a diagnostic term used for eczema up to a specific age in month. | □ |
| --- | --- |

| A diagnosis of infantile eczema is made because a definitive diagnosis cannot be made. | □ |
| --- | --- |
| It is easy to explain the disease and treatment to parents/caregivers | □ |
| A diagnosis of infantile eczema is made for eczema in infancy. | □ |
| Other than the above | □ |

Specify the age in month of patients **at or below** which you often make a diagnosis of infantile eczema.

(enter numerical value).

A diagnosis of "infantile eczema" is often made for patients aged ( ) months **or younger**.

Choose the contents of the explanation to parents/caregivers during the treatment of "infantile eczema."

(check all that apply)

| It commonly develops in infancy. | □ |
| --- | --- |
| Diagnostic term of "infantile eczema" | □ |
| Skin care (cleaning and moisturizing) is important. | □ |
| It resolves spontaneously. | □ |
| It resolves immediately with medication. | □ |
| It requires no medication. | □ |
| It required follow-up. | □ |
| There is nothing to be proactively explained. | □ |
| Others | □ |

Specify the **degree of difficulty in making a diagnosis** of the following skin diseases in infants **in daily clinical practice**

(check one in each row).

|  | Very easy to make a diagnosis | Easy to make a diagnosis | Difficult to make a definite answer | Difficult to make a diagnosis | Very difficult to make a diagnosis |
| --- | --- | --- | --- | --- | --- |
| Seborrhoeic eczema | 1 | 2 | 3 | 4 | 5 |
| Neonatal acne | 1 | 2 | 3 | 4 | 5 |
| Contact dermatitis (diaper rash, drooling rash) | 1 | 2 | 3 | 4 | 5 |
| Nummular eczema | 1 | 2 | 3 | 4 | 5 |
| Asteatotic eczema | 1 | 2 | 3 | 4 | 5 |
| Heat rash | 1 | 2 | 3 | 4 | 5 |
| Atopic dermatitis | 1 | 2 | 3 | 4 | 5 |

For the following diseases, do you often make a diagnosis using a specific disease name, or use a diagnostic term of infantile eczema?

(check one in each row).

|  | In most cases, a diagnostic term of a specific disease is given. | More likely to give a diagnostic term of a specific disease | Case by case | More likely to give a diagnostic term of infantile eczema | In most cases, a diagnostic term of infantile eczema is given |
| --- | --- | --- | --- | --- | --- |
| Seborrhoeic eczema | 1 | 2 | 3 | 4 | 5 |
| Neonatal acne | 1 | 2 | 3 | 4 | 5 |
| Contact dermatitis (diaper rash, drooling rash) | 1 | 2 | 3 | 4 | 5 |
| Nummular eczema | 1 | 2 | 3 | 4 | 5 |
| Asteatotic eczema | 1 | 2 | 3 | 4 | 5 |
| Heat rash | 1 | 2 | 3 | 4 | 5 |
| Atopic dermatitis | 1 | 2 | 3 | 4 | 5 |

Let us know the drugs you prescribe for the treatment of “infantile eczema” in daily medical practice.

(Check all that apply in each column)

|  | Face | Other than face |
| --- | --- | --- |
| Topical moisturizer | □ | □ |
| Topical NSAIDs | □ | □ |
| Non-steroidal topical anti-inflammatory agents (tacrolimus, delgocitinib, and difamilast, etc.) | □ | □ |
| Topical corticosteroids: Strongest (Group I) | □ | □ |
| Topical corticosteroids: Very strong (Group II) | □ | □ |
| Topical corticosteroids: Strong (Group III) | □ | □ |
| Topical corticosteroids: Medium (Group IV) | □ | □ |
| Topical corticosteroids: Week (Group V) | □ | □ |
| Zinc oxide ointment | □ | □ |
| Others | □ | □ |
| No drug is prescribed | □ | □ |

Screen out the responders who checked “No drug is prescribed.”

**From here, let us ask about infantile atopic dermatitis.**

How do you examine infantile patients suspected to have atopic dermatitis at your institution?

(Check one)

| Usually, I examine them by myself at my institution. | 1 |
| --- | --- |
| Usually, another doctor at my institution examines them. | 2 |
| Usually, I refer them to other institutions. | 3 |

The choice of “Usually, another doctor at my institution examines them.” is hidden when there is only one doctor at the institution.

What do you emphasize in making a diagnosis of atopic dermatitis in infants?

(check all that apply)

|  | Not emphasized at all | Not emphasized | Difficult to make a definite answer | Emphasized | Emphasized very much |
| --- | --- | --- | --- | --- | --- |
| Specific age in month or older | 1 | 2 | 3 | 4 | 5 |
| Dried condition | 1 | 2 | 3 | 4 | 5 |
| Itching | 1 | 2 | 3 | 4 | 5 |
| Characteristic rash distribution, such as eczema on both sides of the body | 1 | 2 | 3 | 4 | 5 |
| Confirmed to be chronic and repetitive | 1 | 2 | 3 | 4 | 5 |
| Severe eczema | 1 | 2 | 3 | 4 | 5 |

Specify the age in month of patients **at or above** which you often make a diagnosis of atopic dermatitis in infants.

(enter numerical value).

I often make a diagnosis of atopic dermatitis in infants aged ( ) months **or older**.

What diagnosis do you give to the patient shown below?

Specify the diagnostic term you use in daily medical practice.

(Check one)

| 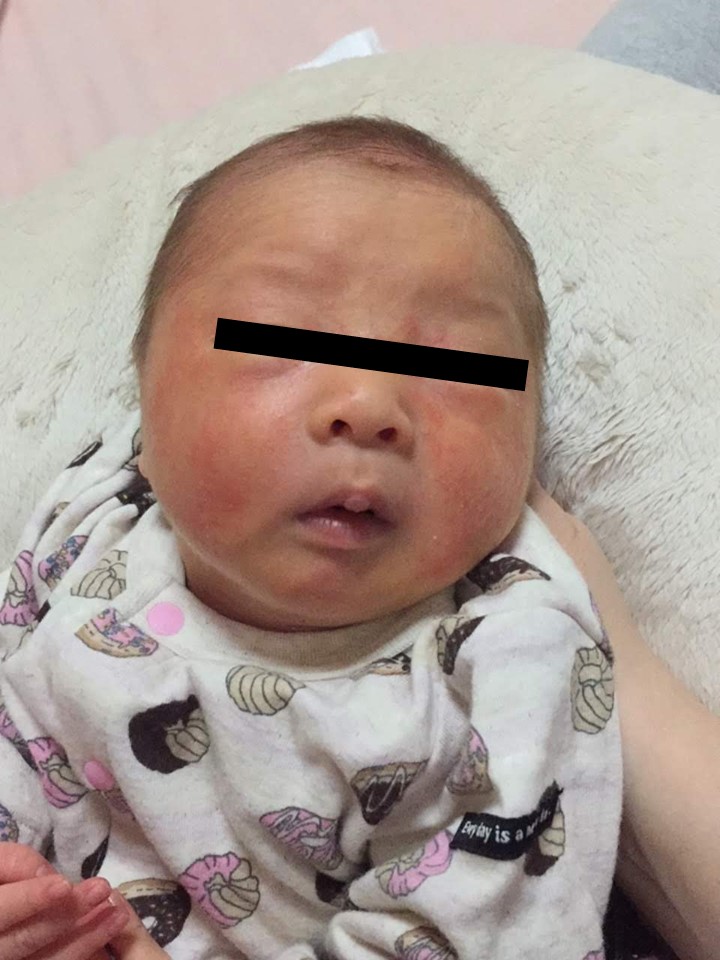  *Case photo: Provided by the medical expert. This photo is presented with the consent of the patient's parent. |
| --- |
| Male infant at 3 months after birth  Rash on the face, as shown in the photo, as early as 1 month after birth It was reported that the face was washed with soap and petrolatum applied once daily. It was also reported that, at the medical checkup at 1 month, the doctor told the mother that rash was common and could be left untreated. When the mother was asked whether the child seemed to have a itch, she replied, “no.” When she was asked whether the child scratched the face when the child was hugged, she answered "sometimes.” Recently, the child had similar rash spreading to the neck.  The child had no skin infection. |

| Infantile eczema | □ |
| --- | --- |
| Atopic dermatitis | □ |
| Seborrhoeic eczema | □ |

What do you explain about infantile atopic dermatitis to the parents/caregivers of patients?

(check all that apply)

| Disease | It is a type of allergic disease. | □ |
| --- | --- | --- |
|  | It resolves spontaneously. | □ |
|  | It resolves as the patient gets older. | □ |
|  | It is important to start treatment early. | □ |
|  | Atopic march | □ |
| Treatment | Skin care (cleaning and moisturizing) is important. | □ |
|  | It improves with short-term treatment. | □ |
|  | It requires long-term treatment. | □ |
|  | It can be remitted with appropriate treatment. | □ |
|  | Adverse drug reactions of prescription drugs, such as topical  corticosteroids | □ |
|  | Therapeutic goal (such as making the disease controllable only with a moisturizing agent) | □ |
| There is nothing to be proactively explained. | | □ |

How do you think the parent/caregiver reacts to the diagnosis of atopic dermatitis in infants? Choose the answer that meets your thinking.

(check one in each row).

|  | Strongly disagreed | Disagreed to some degree | Difficult to make a definite answer | Agreed to some degree | Strongly agreed |
| --- | --- | --- | --- | --- | --- |
| The parent/caregiver may feel relieved because the diagnosis is confirmed. | 1 | 2 | 3 | 4 | 5 |
| The parent/caregiver may become positive for treatment. | 1 | 2 | 3 | 4 | 5 |
| The parent/caregiver may become anxious whether it will be resolved or not. | 1 | 2 | 3 | 4 | 5 |
| The parent/caregiver may not understand what kind of disease the diagnosis means. | 1 | 2 | 3 | 4 | 5 |
| The parent/caregiver may get shocked, considering that the disease is incurable. | 1 | 2 | 3 | 4 | 5 |
| The parent/caregiver may request more explanation on the disease and treatment. | 1 | 2 | 3 | 4 | 5 |

Specify the drugs you prescribe for infantile atopic dermatitis in daily medical practice.

(Check all that apply in each column)

|  | Face | Other than face |
| --- | --- | --- |
| Topical moisturizer | □ | □ |
| Topical NSAIDs | □ | □ |
| Non-steroidal topical anti-inflammatory agents (tacrolimus, delgocitinib, and difamilast, etc.) | □ | □ |
| Topical corticosteroids: Strongest (Group I) | □ | □ |
| Topical corticosteroids: Very strong (Group II) | □ | □ |
| Topical corticosteroids: Strong (Group III) | □ | □ |
| Topical corticosteroids: Medium (Group IV) | □ | □ |
| Topical corticosteroids: Week (Group V) | □ | □ |
| Zinc oxide ointment | □ | □ |
| Antihistamines | □ | □ |
| Others | □ | □ |
| No drug is prescribed | □ | □ |

Screen out the responders who checked “No drug is prescribed.”

Specify the drug you prescribe to the following patient with atopic dermatitis.

Specify the drug you prescribe in daily medical practice.

(Check one)

* If you prescribe more than one drug during daily medical practice, specify one drug most required.

| 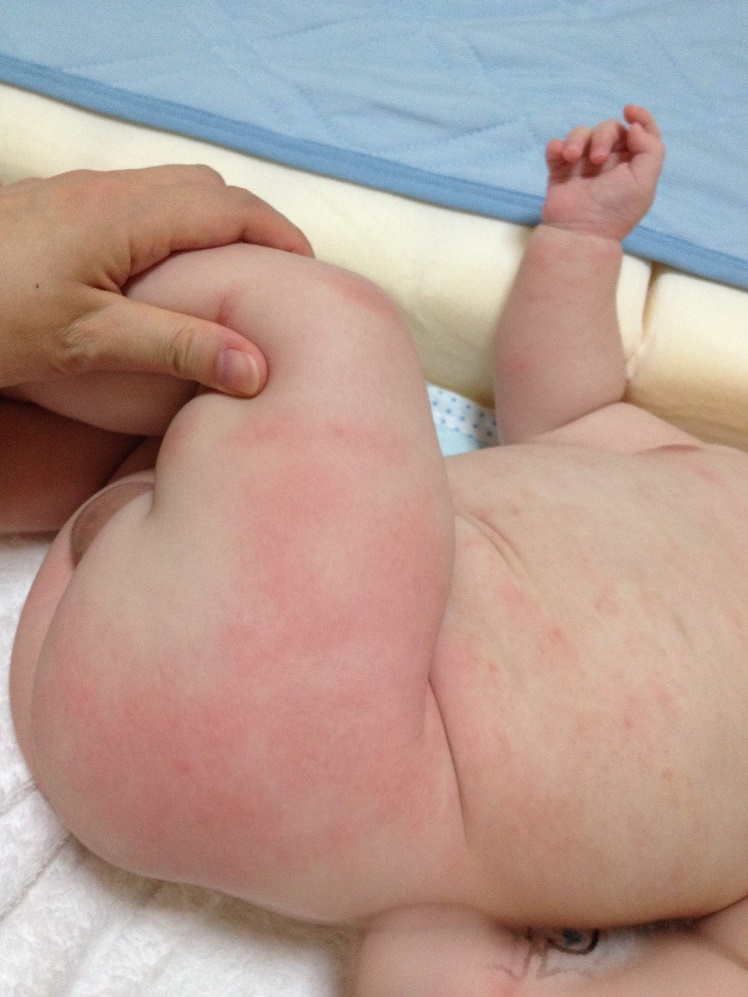  *Case photo: Provided by the medical expert. This photo is presented with the consent of the patient's parent. |
| --- |
| Male infant at 5 months after birth  Papule and erythema developed on the face from 1 month after birth, and rash shown in the photo spread to the neck and body trunk from 2 months after birth. The face and body were washed with soap once daily and only a commercially available moisturizer was used. No diagnosis was made and no prescription given when the patient visited the primary care physician for vaccination. Dry skin was observed at birth, but not now because the skin is treated with a moisturizer every day. It was reported that the child was often bad tempered, although the cause was unknown. No scratching behavior was observed at the office, but it was reported that the child sometimes scratched the body.  The child had no skin infection. |

| Topical moisturizer | 1 |
| --- | --- |
| Topical NSAIDs | 2 |
| Non-steroidal topical anti-inflammatory agents (tacrolimus, delgocitinib, and difamilast, etc.) | 3 |
| Topical corticosteroids: Strongest (Group I) | 4 |
| Topical corticosteroids: Very strong (Group II) | 5 |
| Topical corticosteroids: Strong (Group III) | 6 |
| Topical corticosteroids: Medium (Group IV) | 7 |
| Topical corticosteroids: Week (Group V) | 8 |
| Zinc oxide ointment | 9 |
| Antihistamines | 10 |
| Others | 11 |
| No drug is prescribed | 12 |

Have you felt reluctant to tell the parent/caregiver of a patient with atopic dermatitis the diagnostic term in daily medical practice?

(Check one)

| Frequently | 1 |
| --- | --- |
| Often | 2 |
| Sometimes | 3 |
| Not often | 4 |
| Not at all | 5 |

Choose the most applicable reason for the reluctance to tell the diagnostic term of atopic dermatitis.

(check one in each row).

|  | Not applicable at all | Not applicable much | Slightly applicable | Very applicable |
| --- | --- | --- | --- | --- |
| Because it takes a long time to explain the disease | 1 | 2 | 3 | 4 |
| Because the parent will get shocked | 1 | 2 | 3 | 4 |
| Because the patient may change hospitals | 1 | 2 | 3 | 4 |

Hidden for the respondents who chose "Not at all" in the previous question

Have you ever explained to the parent/caregiver of a patient with atopic dermatitis that **the patient has infantile eczema, not atopic dermatitis**?

(Check one)

| Frequently | 1 |
| --- | --- |
| Often | 2 |
| Sometimes | 3 |
| Not often | 4 |
| Not at all | 5 |

Do you agree the following statement on infantile atopic dermatitis?

If you do not know it, choose "I do not know."

(check one in each row).

|  | I agree | I do not agree |  | I don't know |
| --- | --- | --- | --- | --- |
| The disease resolves spontaneously in most cases | 1 | 2 |  | 3 |
| Eczema can be eliminated with appropriate treatment. | 1 | 2 |  | 3 |
| Early therapeutic intervention is effective. | 1 | 2 |  | 3 |
| Treatment is given for a short period. | 1 | 2 |  | 3 |
| It takes a long time to treat the disease. | 1 | 2 |  | 3 |
| The disease induces other allergic diseases. | 1 | 2 |  | 3 |
| The disease is caused by other allergic diseases. | 1 | 2 |  | 3 |
| Sensitization is established by the oral intake of allergen. | 1 | 2 |  | 3 |
| The oral intake of allergens promotes immune tolerance. | 1 | 2 |  | 3 |
| Sensitization to allergens is established percutaneously. | 1 | 2 |  | 3 |
| The intake of foods causing allergy, such as eggs, should be delayed. | 1 | 2 |  | 3 |

Display: Items randomized

To what degree is atopic dermatitis related to your specialty?

(Check one)

For you, atopic dermatitis is:

| out of specialty. | ← | Difficult to make a definite answer | → | within specialty. |
| --- | --- | --- | --- | --- |
| 1 | 2 | 3 | 4 | 5 |

Choose the answer that comes closest to your thinking about the clinical practice for atopic dermatitis.

(Check one)

| I am anxious about treating atopic dermatitis by myself. | ← | Difficult to make a definite answer | → | I am confident in treating atopic dermatitis |
| --- | --- | --- | --- | --- |
| 1 | 2 | 3 | 4 | 5 |

**From here, let us ask about topical agents to be used in infants.**

When you prescribe a topical agent to an infant, who gives instructions (explains) about how to apply it?

(Check one)

| Doctor | 1 |
| --- | --- |
| Nurse | 2 |
| Pharmacist | 3 |
| Other than the above | 4 |
| No instruction on application is given. | 5 |

Let us know the contents of the explanation by [the explainer reposted] and explanation materials in prescribing topical agents for infants.

**Contents of explanation of application method** (check all that apply)

| FTU (Finger Tip Unit) | □ |
| --- | --- |
| Amount instructed in teaspoonfuls | □ |
| Amount a tissue sticks to | □ |
| Amount looking like shining | □ |
| The patient is instructed to apply an appropriate amount to the affected site. | □ |

**Explanation materials** (check all that apply)

| Video | □ |
| --- | --- |
| Brochure | □ |
| Self-prepared materials | □ |
| Demonstration | □ |
| Others | □ |
| None | □ |

Hidden for the responders who chose “No instruction on application is given” in the previous question.

The “explainer” is indicated when the target of repost is 4. “Other than the above.”

On average, how long does [the explainer reposted] take to explain the application method?

(enter numerical value).

The time required for explanation is ( ) minutes.

Hidden for the responders who chose “No instruction on application is given” in the previous question.

The “explainer” is indicated when the target of repost is 4. “Other than the above.”

What do you do to decide on a treatment policy for infantile atopic dermatitis for return patients? Chose that closest to your thinking for each of the following items.

(check one in each row).

|  | Not at all | Occasionally | Always |
| --- | --- | --- | --- |
| Interview on symptoms | 1 | 2 | 3 |
| Check of the amount of the topical drug applied | 1 | 2 | 3 |
| Check of the frequency of the topical drug applied | 1 | 2 | 3 |
| Check of the site to which the topical drug was applied | 1 | 2 | 3 |
| Appearance check (including the sites covered by clothes) | 1 | 2 | 3 |
| Palpation | 1 | 2 | 3 |

Which of the followings is closest to your thinking for topical corticosteroids used in infancy?

(Check one)

| **May be used** in infants. | 1 |
| --- | --- |
| **May not be used** in infants. | 2 |
| **Not sure if they may be used** in infants. | 3 |

**Lastly, tell us about yourself.**

| **Face sheet** | |
| --- | --- |
| F4. Specialist/advisory doctor authorized by the Japanese Society of Allergology  (check all that apply) | 1. Specialist authorized by the Japanese Society of Allergology  2. Advisory doctor authorized by the Japanese Society of Allergology  3. Neither of the above |
| F5. Age  (Check one) | 1. 20s  2. 30s  3. 40s  4. 50s  5. 60s  6. 70s or older |
| F6. Experience in the treatment of infantile eczema  (enter numerical value). | About ( ) years |
